# Supplementary material for: OsPP2C09 Is a Bifunctional Regulator in Both ABA-Dependent and Independent Abiotic Stress Signaling Pathways
Source: Int J Mol Sci. 2021 Jan 1;22(1):393. doi: 10.3390/ijms22010393 (PMC7795834; doi:10.3390/ijms22010393)
Supplement: Supplementary file 1 [file ijms-22-00393-s001.pdf]

International Journal of *Molecular Sciences*

Supplemental Methods

Drought Treatments in Soil

For drought treatments, about 20 plants from each overexpression line were grown in pots (L165 × W80 × H70 mm) filled with soil alongside wild type as control. We stopped watering when seedlings were 14 days old, and watering was resumed for 7 days one week later, after which the survival rates were calculated.

Subcellular Localization

To observe the subcellular localization of GFP-fusion proteins, the indicated genes were inserted into pENTR/D-topo vectors (Invitrogen, Carlsbad, CA, USA). The coding sequences were recombined into the pMDC83 vector via LR recombination reaction using the Gateway system (Invitrogen, Carlsbad, CA, USA) [35]. The constructs were introduced into rice protoplasts together with NLS-RFP or alone using the PEG-mediated method. The protoplasts transformed with a single construct were fixed with paraformaldehyde (4%) and then stained with DAPI before observation. The signals of GFP, RFP and DAPI were captured using a Leica TCS SP8 laser scanning confocal microscope. The combinations of excitation wavelength/detection range of emission on the confocal microscopy were 488 nm (solid state laser)/493 to 530 bandpass for GFP. The detected images are presented in pseudo-colors. To quantify the rate of co-localization between two different proteins in the same image, images were analyzed with the Leica Application SuitX analysis program using the default settings (threshold 30%, background 20%).

Yeast Two-Hybrid Assay

Yeast two-hybrid assays were performed using the Matchmaker™ GAL4 Two-Hybrid System 3 (Clontech, Mountain View, CA, USA), according to the manufacturer's manual. The lithium acetate method was used to introduce *AD-OsPP2Cs* and *BD-OsPYL/RCAR3* into *Saccharomyces cerevisiae* strain AH109 [36]. Yeast cells were grown on Yeast Minimal Medium/Synthetic Defined (SD) medium (Clontech, Mountain View, CA, USA) lacking leucine and tryptophan, then transferred to selection medium lacking leucine, tryptophan, and histidine, supplemented with 2 mM 3-amino-1,2,4-triazole (3-AT) (Sigma-Aldrich, St. Louis, MI, USA). Exponentially growing yeast cells were harvested and adjusted to OD_{600} = 0.5 using sterilized water, and then yeast cells were plated on SD medium without leucine and tryptophan, and the SD medium without leucine, tryptophan, or histidine. Their growth was observed after three days.

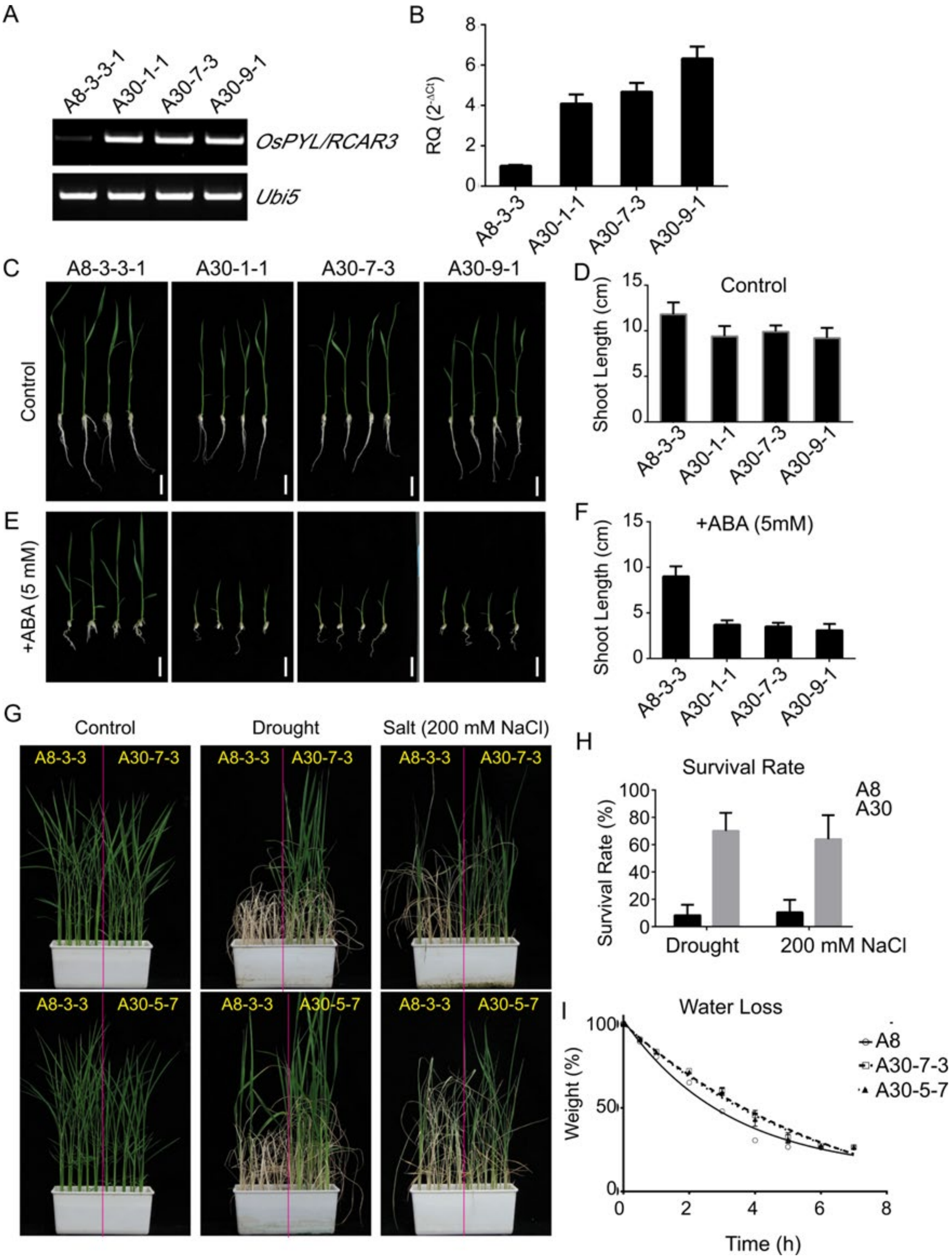

Figure S1. A30 (overexpressing OsPYL/RCAR3) plants and their tolerance to abiotic stresses. (**A**), RT-PCR amplification of *OsPYL/RCAR3* in wild-type and A30 plants. (**B**), RT-qPCR of *OsPYL/RCAR3* in wild-type and A30 plants. (**C**–**F**), Seeding growth test with or without ABA. The values are means of each length of over 10 plants and error bars are SD. (**G**,**H**), Tolerance test for drought and salt stress.

A

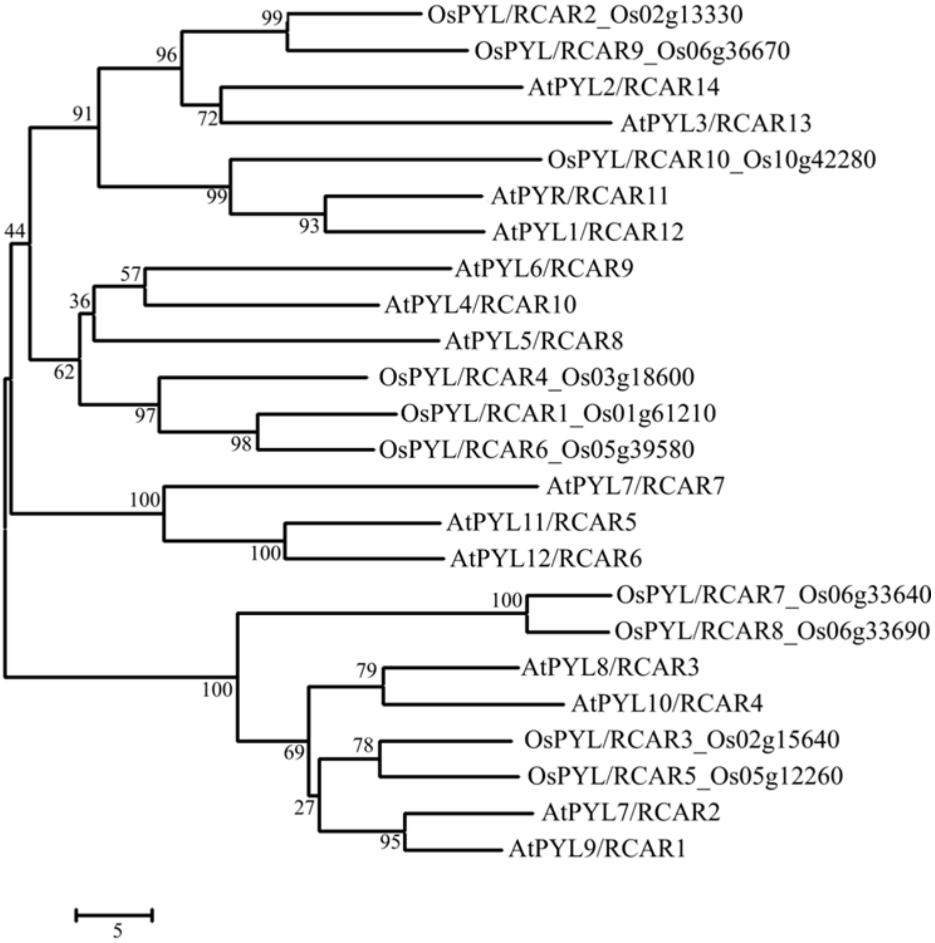

B

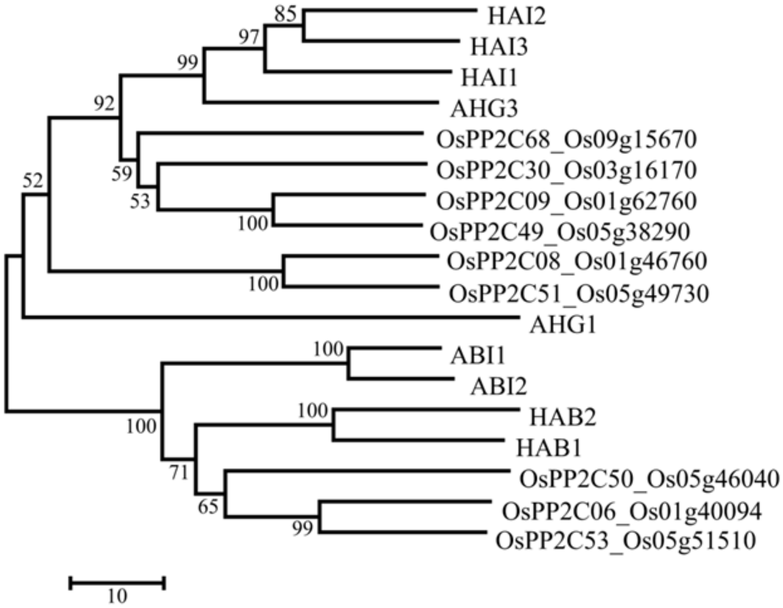

Figure S2. Phylogenetic trees of ABA receptors (**A**) and PP2CAs (**B**) from rice and Arabidopsis.

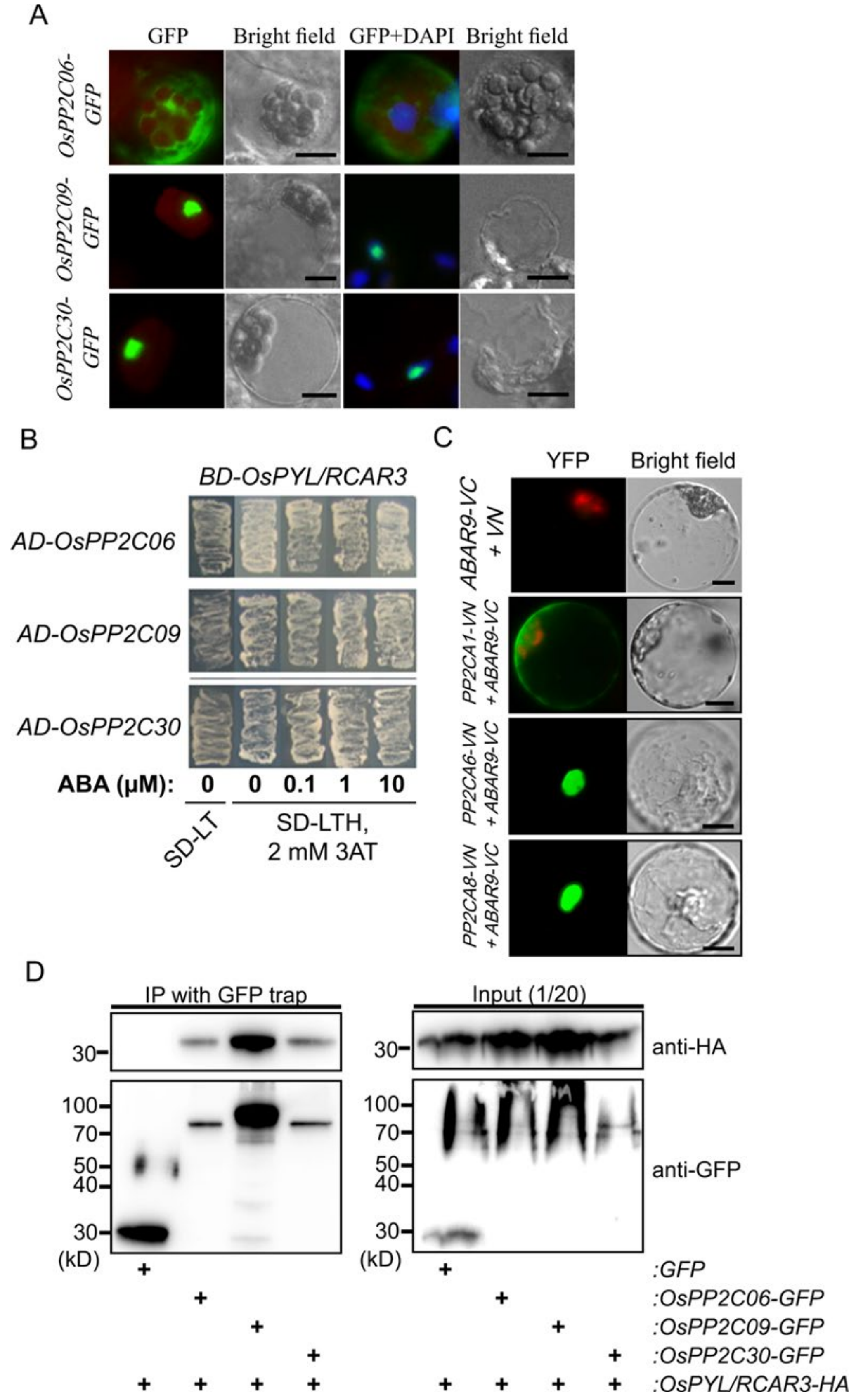

Figure S3. ABA-independent interaction of OsPYL/RCAR3 and OsPP2Cs. (**A**), Localization of OsPP2Cs in rice protoplasts. Scale bars: 10 μM. (**B**), Yeast two-hybrid assays. (**C**), Bimolecular fluorescence complementation assay in rice protoplasts. Scale bars: 10 μm. (**D**) Co-immunoprecipitation. The indicated proteins were transiently expressed in rice protoplasts and pulled-down with GFP-trap beads. All experiments were repeated more than three times with similar results.

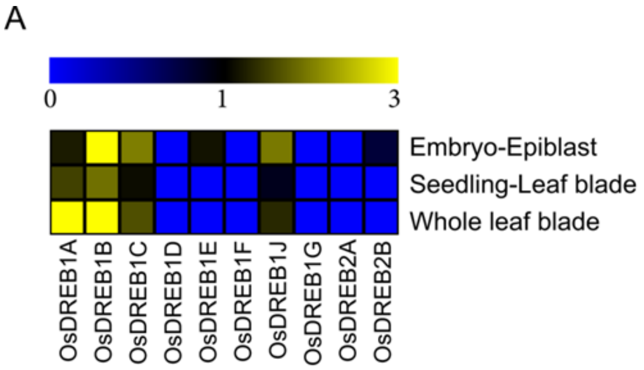

B

GEO number	Description
GSM329536<br>GSM329537<br>GSM329538<br>GSM329539	Embryo - Epiblast
GSM329628<br>GSM329629<br>GSM329630<br>GSM329631	Seedling - Leaf blade
GSM329656<br>GSM329657<br>GSM329658<br>GSM329659	Tissue - Whole leaf blade

Figure S4. Search for *OsDREBs* expression in the rice microarray data base. (**A**), Heatmap of *OsDREB* expression. (**B**), Used GEO numbers and descriptions.

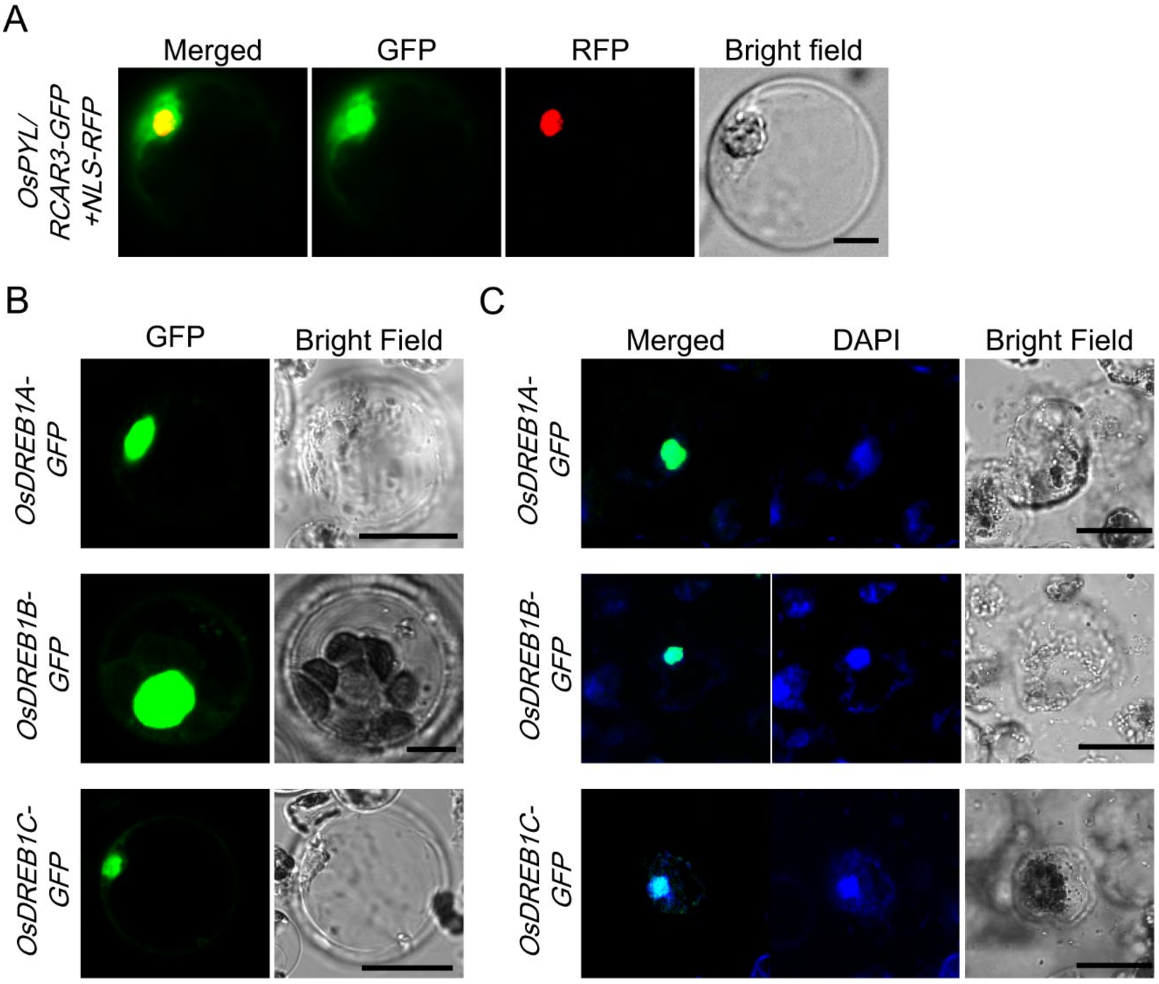

Figure S5. Subcellular localization of OsPYL/RCAR3 and OsDREB1s. (**A**), OsPYL/RCAR3-GFP co-localize with NLS-RFP, a nuclear marker. Scale bar: 10 μm. (**B**), Subcellular localization of OsDREB1A-GFP, OsDREB1B-GFP, and OsDREB1C-GFP. Scale bar: 10 μm. (**C**), OsDREB1sGFP signal was merged with DAPI staining, performed after fixation with 10 mM PFA. Scale bar: 10 μm.

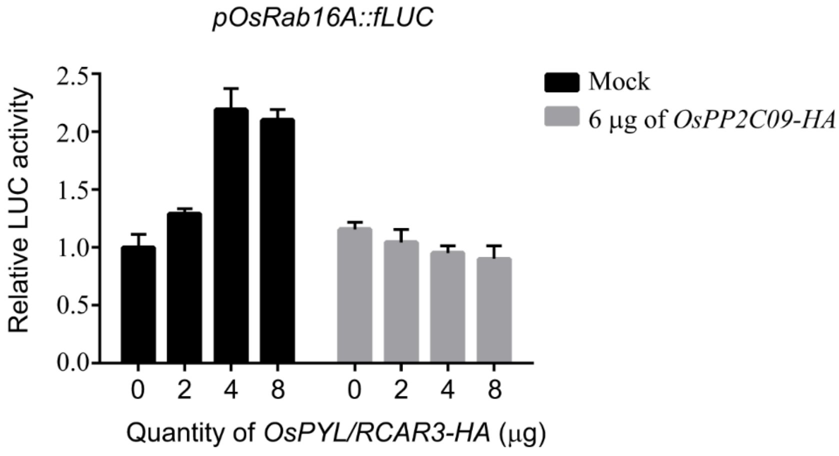

Figure S6. Effects of OsPYL/RCAR3 overexpression on OsPP2C-mediated induction of luciferase activity from the *pOsRab16A::fLUC* reporter. The indicated effector and marker DNAs were introduced into rice protoplasts by PEG-mediated transfection and then incubated for 15 h. Induced luciferase activity was detected with a dual-luciferase reporter assay system. All values are means ±SD (n = 3).
